# Development of the alcohol‐free and low‐alcohol drinks market in Great Britain from 2011 to 2022: Narrative timelines based on a documentary review of off‐trade retail magazines and market intelligence reports

**DOI:** 10.1111/dar.14058

**Published:** 2025-05-05

**Authors:** Nathan Critchlow, Amber Morgan, Kathryn Angus, Rebecca Howell, Niamh Fitzgerald, Inge Kersbergen, John Holmes

**Affiliations:** ^1^ Institute for Social Marketing and Health University of Stirling Stirling UK; ^2^ Sheffield Addictions Research Group University of Sheffield Sheffield UK

**Keywords:** alcohol‐free and low‐alcohol, documentary review, market development, narrative timelines, zero alcohol

## Abstract

**Issue:**

There is a growing alcohol‐free and low‐alcohol (no/lo) drinks market in Great Britain. Insight about when this emerged and how it has developed is needed to inform and interpret the growing body of research into the use of no/lo drinks. We therefore document the development of the no/lo market in Great Britain between 2011 and 2022 and examine which stakeholders have been involved in development and what actions they have taken.

**Approach:**

Narrative timelines created through a documentary review of trade magazines (2011–2022) and market intelligence reports (2015–2022), focusing on product launches, marketing activity, industry changes, retailer actions, governmental actions and third sector activity.

**Findings:**

A mainstream no/lo market emerged and established from 2015, with activity thereafter characterised by intensive market entry, expansion, and consolidation among both independent producers and mainstream alcohol brands. While initial development concentrated on beers, innovation has since proliferated across the cider, spirits, wine and ready‐to‐drink categories. Development appears predominately driven by market forces (e.g., product launches and marketing), with January a focal point of activity. Government has not introduced any legislation around no/lo drinks, although it has consulted on appropriate no/lo descriptors (in 2018) and committed (in 2019) to work with industry to grow the no/lo market.

**Implications and Conclusions:**

While initial development in the no/lo market concentrated on beers, recent developments across categories, coupled with continued consolidation and expansion among beers, suggest the market may still develop further. Any assessment of the public health impact of no/lo drinks should be subject to longer‐term follow‐up once the market matures.

## 1 INTRODUCTION

In Great Britain (GB), alcohol‐free and low‐alcohol drinks (‘no/lo’) are defined as beers, ciders, spirits and wines containing <1.2% alcohol‐by‐volume (ABV) [1]. The no/lo drinks market in GB has grown substantially in recent years [2]. Retailers sold 1.5 litres of no/lo drinks per adult in GB in 2023 [3], with 31.3% of adults in GB reporting ever consuming no/lo drinks and 9.8% doing so on a weekly basis [4]. Despite this, no/lo drinks still only represent a small proportion of the overall alcoholic drinks market in GB, accounting for 1.4% of sales volume and 0.8% of sales value in 2023 [3].

Increased availability and consumption of no/lo drinks in GB has led to policy interest in whether these drinks could reduce consumption of standard‐strength alcohol and subsequent associated harms [5]. There are, however, some uncertainties and risks. For example, consumers may only add no/lo drinks to their consumption of standard‐strength alcohol—addition rather than substitution—thus leading to no meaningful decline in alcohol use. Moreover, public health improvements may only be small if uptake mostly occurs among those already drinking at lower‐risk levels, while health inequalities may widen if consumption continues to concentrate among those from more affluent backgrounds [4]. Finally, exposure to no/lo drinks which share branding with standard‐strength alcoholic drinks may encourage consumption of both types, and some groups may be particularly susceptible to the effects of shared branding (e.g., young people or those abstaining from alcohol) [6]. Young people may also develop earlier familiarity with the taste of alcohol, which may lead to earlier or greater consumption of standard‐strength alcohol.

To date, however, there is limited research which has examined the development of the no/lo drinks market in any country. This leaves important questions unanswered, such as when did the market emerge, is development ongoing, what product categories have engaged with the market, and what role have different stakeholders played in market development. These insights are needed to help inform and interpret the growing body of research into no/lo drinks, such as analyses of consumer trends [2, 3, 4]. We, therefore, document the development of the no/lo drinks market in GB between 2011 and 2022, overall and within specific drink categories, and examine which stakeholder groups have been involved in market development (e.g., industry, retailers and government) and what actions they have taken (e.g., product launches, marketing activity or regulatory changes).

## 2 METHODS

### 2.1 Design

We created narrative timelines of events which have contributed to development of the no/lo drinks market in GB between 2011 and 2022. Data come from a documentary analysis of market intelligence reports and magazines aimed at off‐trade retailers (hereafter the ‘retail trade press’). These industry‐focused data sources provide novel insight into market development for addiction and health‐related commodities, and have been used previously to examine the tobacco, alcohol and food/drink markets [7, 8, 9, 10, 11, 12]. This study is part of a larger project evaluating the public health impact of no/lo drinks in GB [13].

### 2.2 Data sources

Table 1 provides an overview of the two data sources used. For market intelligence reports, we conducted a scoping exercise to identify reports from leading suppliers relating to both the wider GB alcoholic drinks market and specifically the no/lo drinks market. From this, we purchased five reports by Mintel entitled *‘Attitudes towards low‐ and non‐alcoholic/no‐alcohol drinks’*, published between 2015 and 2022.

**TABLE 1:** Summary of the data sources reviewed.

Publication	Focus and target readership	Years included	Frequency
The Grocer	General grocery market	January 2011–December 2022	Weekly
Forecourt Trader	Fuel retail sector (garages/forecourts)	January 2011– December 2022	Monthly until September 2020 and every other month thereafter
Off Licence News, renamed as Drinks Retailing News and latterly to Drinks Retailing	Specialist publication aimed at off‐trade alcohol outlets	January 2011–December 2022	~Twice monthly 2011–2015 ~Monthly 2016–2019 Every other month 2020–2022
Convenience Store	Smaller and independent retailers	January 2011–April 2020^a^	Twice monthly
RN (Retail Newsagent)	Smaller and independent retailers	March 2017^b^–December 2022	Weekly
Mintel *‘Attitudes towards low‐ and non‐alcoholic/no‐alcohol drinks ‐ UK’*	Specialist market intelligence report on the alcohol‐free and low‐alcohol (no/lo) market	2015–2022	Published in 2015, 2017, 2019, 2021, and 2022

^a^ Title moved to online only from April 2020 and thus ceased to be included in the study.

^b^ Title only added to the library from March 2017, and thus earlier issues are not available.

For the retail trade press, we reviewed paper copies of five magazines aimed at off‐trade retailers in GB published between 2011 and 2022. By off‐trade retailers, we mean shops where alcohol can be purchased for consumption off the premises, such as supermarkets, convenience stores, and off‐licences. These magazines typically contain news, opinion, and feature articles on market industry trends and developments, as well as advertisements from producers. Articles typically cover a range of fast‐moving consumer goods, including alcoholic drinks, as well as articles of general interest to retailers. We focused on magazines aimed at the off‐trade market for no/lo drinks because it is substantially larger and more developed than its on‐trade equivalent (i.e., venues where alcohol is purchased for consumption on the premises, such as pubs or bars) [3].

The trade press magazines were sampled from an existing library of paper magazines held by the authors. This library has been purposively curated for almost two decades to include a combination of leading trade press titles aimed at different parts of the retail sector in GB, including general grocery retailers, off‐licences, convenience stores, newsagents, and garage forecourts or petrol stations. We included all the titles available in the library across our observation period (~1300 magazines; see Table 1 for publication dates, frequencies, and focus). We preferred to utilise paper copies of the magazines, as opposed to searching online databases which include similar titles, as the latter often omit important content, such as advertisements, advertorials, graphics, and images. All paper copies were manually reviewed, with all content related to no/lo drinks read line‐by‐line.

Where necessary, we used purposive ad‐hoc searches of open‐source data (e.g., policy reports, online news articles) to corroborate or embellish details of events reported in either the market intelligence reports or trade press magazines.

### 2.3 Products in scope

To capture all potentially relevant information, we manually searched each data source for all references to drinks that were suggested to be part of, or related to, the no/lo market. In practice, this meant searching for a range of evolving terms, including, but not restricted to: ‘no‐alcohol’, ‘non‐alcoholic’, ‘alcohol‐free’, ‘de‐alcoholised’, ‘zero‐alcohol’, ‘low‐alcohol’, ‘lower‐alcohol’, ‘moderation markets’ and ‘alcohol‐alternatives’. These are hereafter referred to collectively as ‘no/lo drinks’, unless quoting or paraphrasing a source. We also searched for content related to non‐alcoholic drinks which incorporated alcohol connotations, such as soft‐drinks which mimicked alcoholic drinks (e.g., cocktail‐flavoured) or drinks supplied in packaging considered to mirror characteristics typically associated with alcohol (e.g., champagne‐style bottles).

### 2.4 Defining events

Across sources, we sought to identify and capture information on six ‘event’ categories, derived from initial scoping of the data sources. These were: (i) product launches, including rebrands or relaunches; (ii) marketing activity, such as advertising campaigns; (iii) industry changes, such as mergers or acquisitions; (iv) retailer actions, such as changes in product listings or point‐of‐sale promotional activity; (v) government actions; and (vi) activity from third sector organisations, such as abstinence campaigns run by charities. This review did not seek to directly capture generic discussion of the no/lo market which did not refer to a specific event, such as commentary on sales trends. We also did not aim to capture information on the market or corporate political strategy of companies in relation to no/lo drinks, such as their motives for market entry or longer‐term market strategies. Both aspects are covered in‐depth elsewhere in the overall project [13].

### 2.5 Recording events

Details about each event were recorded in a codebook created in Microsoft Excel. Each event was recorded as a separate data row, and we maintained separate worksheets for each year. Events were captured each time they were reported in one of the data sources, irrespective of whether they had been mentioned previously (e.g., the same event described in multiple trade press magazines and the market intelligence reports). For each event, we recorded: (i) bibliographic information; (ii) approximate timing; (iii) detailed and short summaries; and (iv) which companies (e.g., Diageo), brands (e.g., Guinness 0.0%), and product types (e.g., beers) were mentioned. We also coded whether the event related to any of the six event categories. An event could be coded under multiple categories (e.g., product launch with associated marketing campaign).

### 2.6 Analysis

Data were analysed in three stages. First, we used the detailed extraction database to create a separate database in which each row represented an individual event in each year, thus consolidating repeat entries of the same event. This enabled us to build a detailed picture of each event, as different sources provided varying degrees of detail, and to see which events were discussed repeatedly, thus providing insight into their potential importance in shaping the no/lo drinks market. We then used this refined database to create detailed timelines for the main no/lo drink categories: beer, cider, pre‐mixed/ready‐to‐drink, spirits, wines, and adult soft drinks. We organised our analysis around these categories to reflect that they have developed at different paces and to different degrees across the observation period. We also created a separate timeline for cross‐cutting events which may have affected the overall no/lo drinks market. Finally, we used this refined database to produce narrative summaries and visual timelines which chart the key development phases for each category and examples of events within each phase. Where relevant, the narrative summaries reference categories adjacent to the no/lo market (e.g., ‘lower strength’ alcohol). The visual figures, however, only report events related to no/lo drinks.

## 3 RESULTS

The results begin with cross‐cutting events likely to have affected the overall no/lo market. We then present the category‐specific timeline for beer, where early market development concentrated, before presenting timelines for categories that emerged and developed later. For each section, we provide a visual timeline of key phases and example events, and a narrative summary of market development.

### 3.1 Cross‐cutting events

**FIGURE 1:**
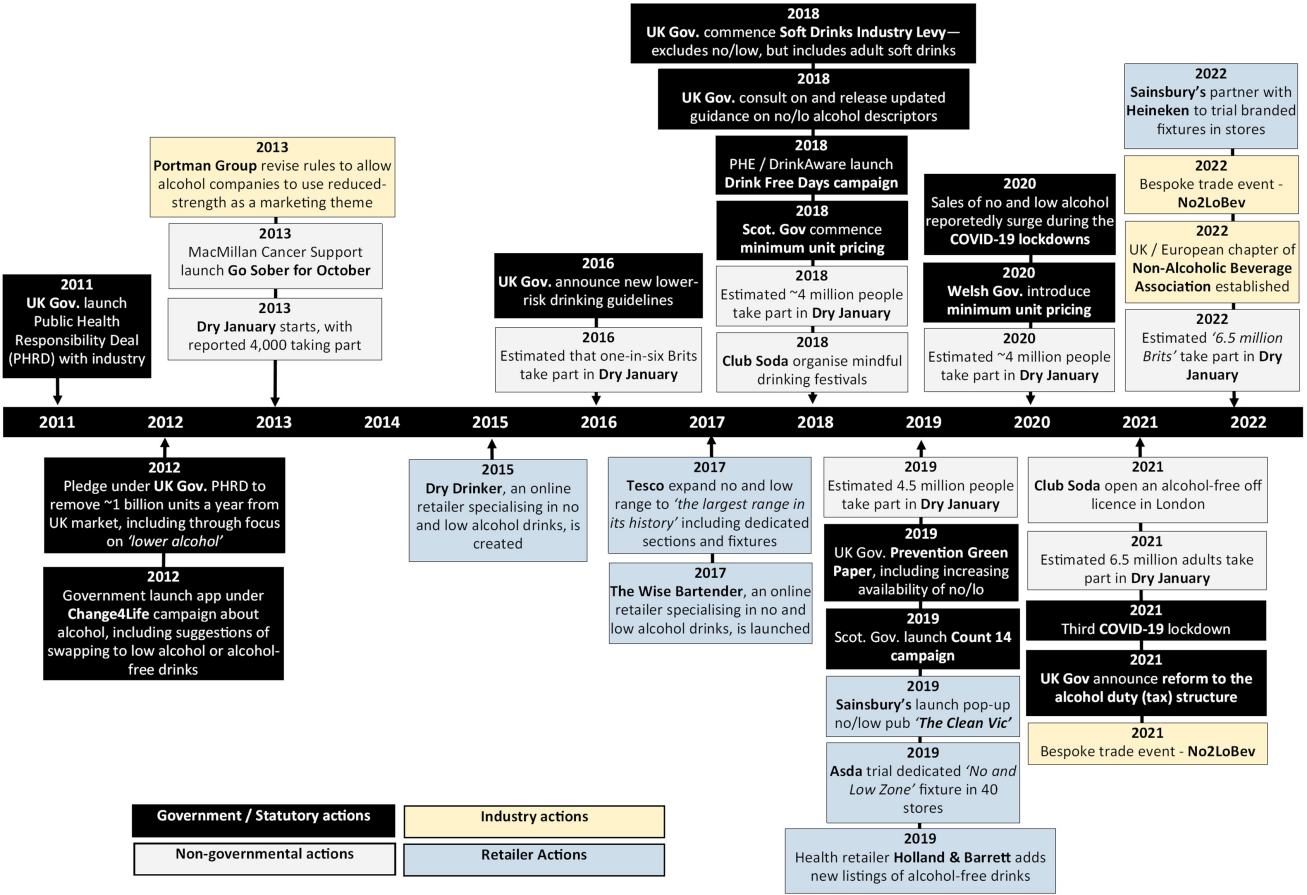
Timeline of cross‐cutting events which may have directly or indirectly impacted the overall no/lo market between 2011 and 2022.

Figure 1 summarises cross‐cutting events likely to have directly or indirectly affected the overall no/lo drinks market. The frequency and volume of such events increased in the latter half of the timeline as government and industry began to consider regulatory issues for no/lo drinks, retailers became increasingly engaged in the market, and the market interacted with third sector activity.

As will be discussed in the category‐specific timelines, most of the events we observed were led by supply‐side stakeholders, such as product launches and associated marketing campaigns. Nevertheless, we identified two activities from the UK Government that were deemed directly relevant to the no/lo drinks market. First, in March 2018, the Government launched a consultation on the appropriate use of no/lo descriptors, a set of terms (e.g., alcohol‐free, non‐alcoholic, de‐alcoholised, etc.) defined and recommended for use by Government, but not legally mandated [14, 15]. This consultation led to revised guidance on descriptors being published in December 2018 [1, 16]. Second, in 2019, the UK Government included a commitment in a policy paper to work with the industry to deliver a ‘*significant increase*’ in the availability of no/lo drinks by 2025 [5], although this was not accompanied by—or, to date, followed‐up with—any specific policies.

There were also several cross‐cutting government events which may have indirectly influenced the no/lo market. A key early event was the Public Health Responsibility Deal (PHRD), launched in 2011 [17, 18]. Under the PHRD, ~30 major producers and retailers pledged in March 2012 to remove one billion units of alcohol from the market each year by December 2015, including by improving the availability and promotion of ‘*lower alcohol*’ drinks [18, 19, 20]. The PHRD is only considered indirectly relevant to the no/lo market as the discourse and related initiatives predominately focused on products that were only lower‐strength relative to their traditional counterparts (e.g., 2–3% ABV beers or 5.5% ABV wines) rather than no/lo drinks under our definitions of <1.2% ABV (albeit a very small number of no/lo products were launched towards the end of this period; see examples in category timelines). Other government actions with the potential to indirectly affect the no/lo market included launching revised low‐risk drinking guidelines in January 2016 [21, 22], public health awareness campaigns around reduced alcohol use [16, 22, 23, 24], and increased off‐trade sales of no/lo drinks during the government‐mandated COVID‐19 ‘lockdowns’ [25, 26]. The Government also announced a reform of the duty system for alcoholic drinks in October 2021, with the new approach broadly taxing all drinks in relation to their strength and simplifying the structure and administration of the duty system [27]. Although the reforms did not take effect until autumn 2023, after the end of our study period, it was reported that this disincentive to produce higher strength products may *‘encourage further innovation’* for no/lo drinks [27].

For retailers, cross‐cutting events mostly took the form of new and expanded listings of no/lo drinks (i.e., range and number of products stocked), particularly across the latter half of the timeline. Supermarket chain Tesco were a key early adopter, expanding their no/lo drinks offering to *‘the largest range in its history’* in March 2017 and stating their aim to *‘set the gold standard on low/no choice within grocery’* [22, 28]. This was followed by activities from other major retailers, including a pop‐up alcohol‐free bar from Sainsbury's [29] and trials of dedicated no/lo fixtures in ASDA [30], Sainsbury's (in partnership with Heineken) [31] and Holland & Barrett (a health supplement retailer) [32]. There was also evidence of an online market developing for no/lo drinks, such as the launch of the retail websites Dry Drinker in 2015 [33] and Wise Bartender in 2017 [34].

Most producer activity related to specific no/lo categories and is covered in subsequent sections of this paper. Nevertheless, there were several cross‐cutting events from producers pertinent to the overall market. A key early development was a revision to the marketing code produced by the Portman Group, an industry self‐regulatory body in GB, as guidance for its members. The code had previously prevented ‘higher’ or ‘lower’ levels of alcohol being used as a dominant theme in the marketing of alcoholic drinks. From May 2013, however, the code was revised to reportedly ‘*remove the barriers which previously prevented producers from promoting low and lower‐alcohol alternatives*’ [35, 36], providing such marketing claims were made in a ‘*proportionate* *manner*' [37]. The growing prominence of the no/lo market in the latter stages of the timeline was also reflected in the launch of a UK trade show dedicated to the sector, called ‘*Low2NoBev*’ [38, 39] (first held in 2021), and establishment of a UK and European chapter of the ‘*Adult Non‐Alcoholic Beverage Association*’ in 2022 [40].

The final cross‐cutting events related to third sector organisations. Central to this was increased public engagement in (temporary) abstinence campaigns, particularly Dry January®, which is run by Alcohol Change UK (formerly Alcohol Concern). The proportion of people going ‘dry’ during January increased across the timeline, from a reported 4000 in 2013 [41] to an estimated 6.5 million by 2022 [42]. Although attempts at healthier living have long been associated with January, increased consumer engagement in taking a break from alcohol provided an opportunity for producers to make this month a focal point for promoting no/lo drinks in the latter stages of the timeline. Specifically, product launches and marketing activity often appeared to concentrate around January [41] and some brands agreed endorsement deals with the official Dry January® campaign [42]. Separately, there was also regular activity from Club Soda, a social impact business described as ‘*one of the UK's leading proponents of mindful drinking*’ [43]. This included organising festivals to promote mindful drinking in 2018, which included focus on no/lo drinks [43], and opening an alcohol‐free off‐licence in 2021 [44].

### 3.2 Beer

**FIGURE 2:**
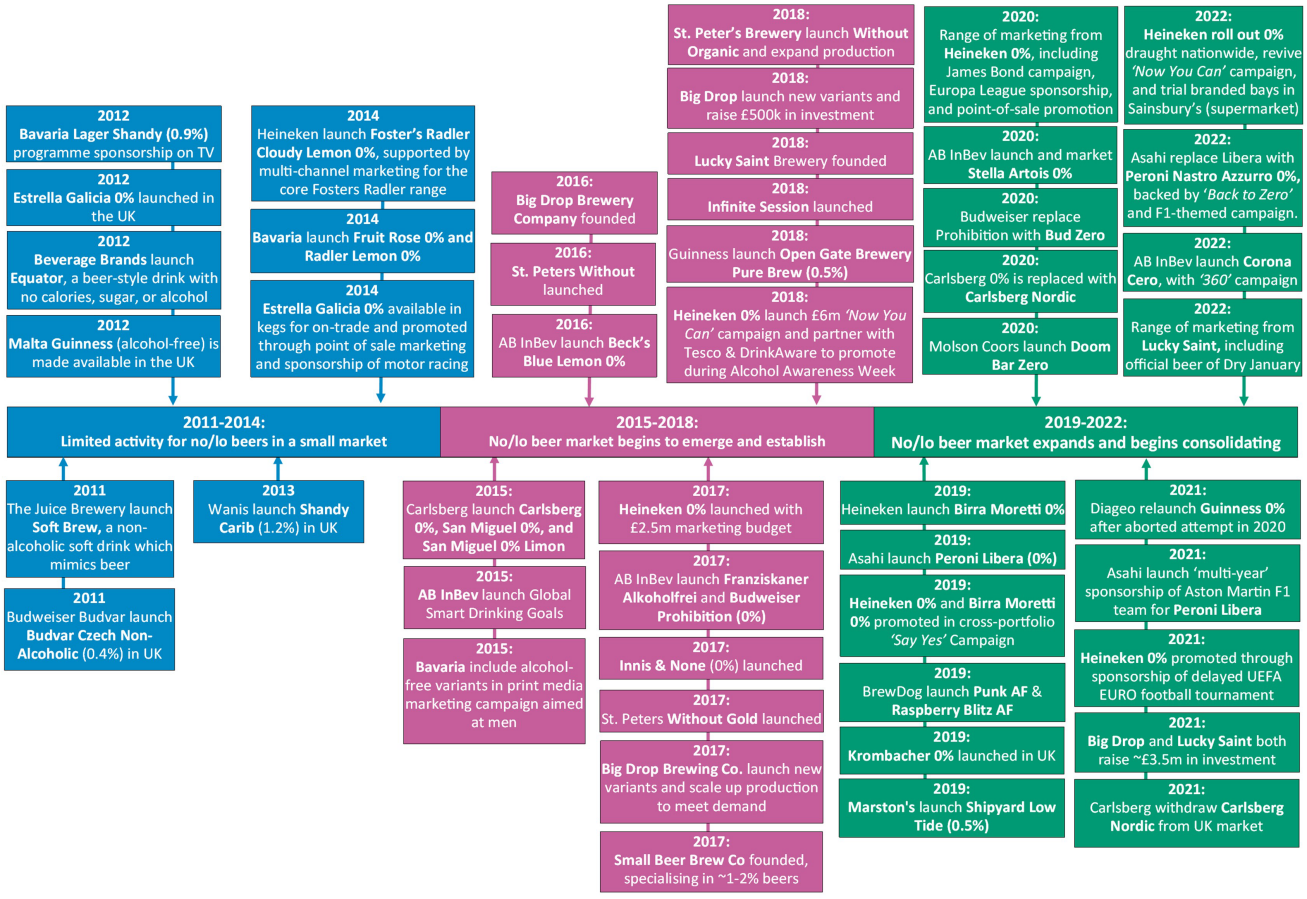
Timeline of key phases and example events in the development of the no/lo beer market between 2011 and 2022.

At time of writing, beer is by far the largest sector of the no/lo market in GB, accounting for ~70% of sales value in 2023 [3]. Figure 2 illustrates the three key phases and example events observed in its development. In phase one (2011–2014), activity was limited in both scope and scale and mostly did not involve mainstream producers. The focus of mainstream producers was instead mostly towards lower‐strength beers, particularly those with fruit/citrus flavours. This activity occurred in parallel to the PHRD pledge to increase the availability of lower‐strength products [18, 19, 20] and Government cuts to the duty payable on beers <2.8% ABV in 2011 [45] and 2013 [46]. Notable ‘lower‐ABV’ launches from mainstream brands, often backed by substantial marketing support, included: Carling Zest (2.8%) [47], Carlsberg Citrus (2.8%) [48] and Foster's Radler (2%) [49]. This focus on lower‐strength beers did, however, eventually lead to interest from some mainstream producers in no/lo drinks. Heineken, for example, introduced an alcohol‐free variant of Foster's Radler in 2014, with the variant included as part of marketing for the main Radler range [50, 51].

In the second phase (2015–2018), activity for lower‐ABV beers slowed and the no/lo market began to emerge and establish, including launches from mainstream brands. Carlsberg, for example, launched an alcohol‐free version of their core brand [52] and two alcohol‐free San Miguel brand variants in 2015 [53]. Moreover, AB InBev launched their Global Smart Drinking Goals at the end of 2015, which included a commitment to make at least 20% of their global beer volume ‘*no or lower‐alcohol*’ by the end of 2025 [54], and launched both a lemon variant of alcohol‐free Beck's Blue in 2016 [55] and alcohol‐free Budweiser Prohibition in 2017 [56]. A key event during this second phase was the launch of Heineken 0.0% in 2017 [57], which quickly became the best‐selling alcohol‐free beer. The product was backed by a £2.5 million marketing investment on launch [58] and was further supported by the £6 million ‘*Now You Can*’ campaign in 2018 [59]. This second phase also saw the emergence and growth of the independent no/lo beer market. For example, Big Drop Brewing Company was founded in 2016 [60], and the company both scaled up production in 2017 to meet demand [60] and raised £500k of investment funds in 2018 to aid its growth [61]. St. Peter's Brewery also launched alcohol‐free variants in 2016, 2017 and 2018 [28, 62, 63], and reported increasing its production to keep pace with demand [64].

The final phase (2019–2022) was characterised by further market entry from mainstream beer brands, including many current market leaders, and consolidation among those already active in the market. For new entrants, Asahi UK launched alcohol‐free Peroni Libera in 2019 [65] and replaced it with a variant using the core Peroni Nastro Azzurro branding in 2022 [66], with the relaunch supported by the ‘*Back to Zero*’ campaign [67]. Diageo also formally launched Guinness 0.0% in 2021 [68], backed by a ‘*massive consumer campaign*’ [68], following an unsuccessful launch a year earlier that was curtailed by a product recall [69]. In terms of consolidation, Heineken 0.0% sought to strengthen their position through extensive marketing. This included football sponsorship [70], leveraging their partnership with the James Bond film franchise [24], point‐of‐sale promotion [31], and reviving the ‘*Now You Can*’ campaign in January 2022 [67]. AB InBev also consolidated their position by expanding their portfolio to include alcohol‐free versions of Stella Artois and Corona (Cero), both reportedly backed by marketing support [71, 72], and replacing Budweiser Prohibition with Budweiser Zero [73]. This final phase also saw continued growth and expansion among independent producers. For example, both Lucky Saint and Big Drop Brewing Co. reportedly raised £3.5 million in investment in 2021 [74, 75], while Big Drop reportedly raised a further £2.3 million in crowdfunding support in 2022 [76] and Lucky Saint was named ‘*the first ever official beer of Dry January*’ in 2022 [42].

### 3.3 Cider

**FIGURE 3:**
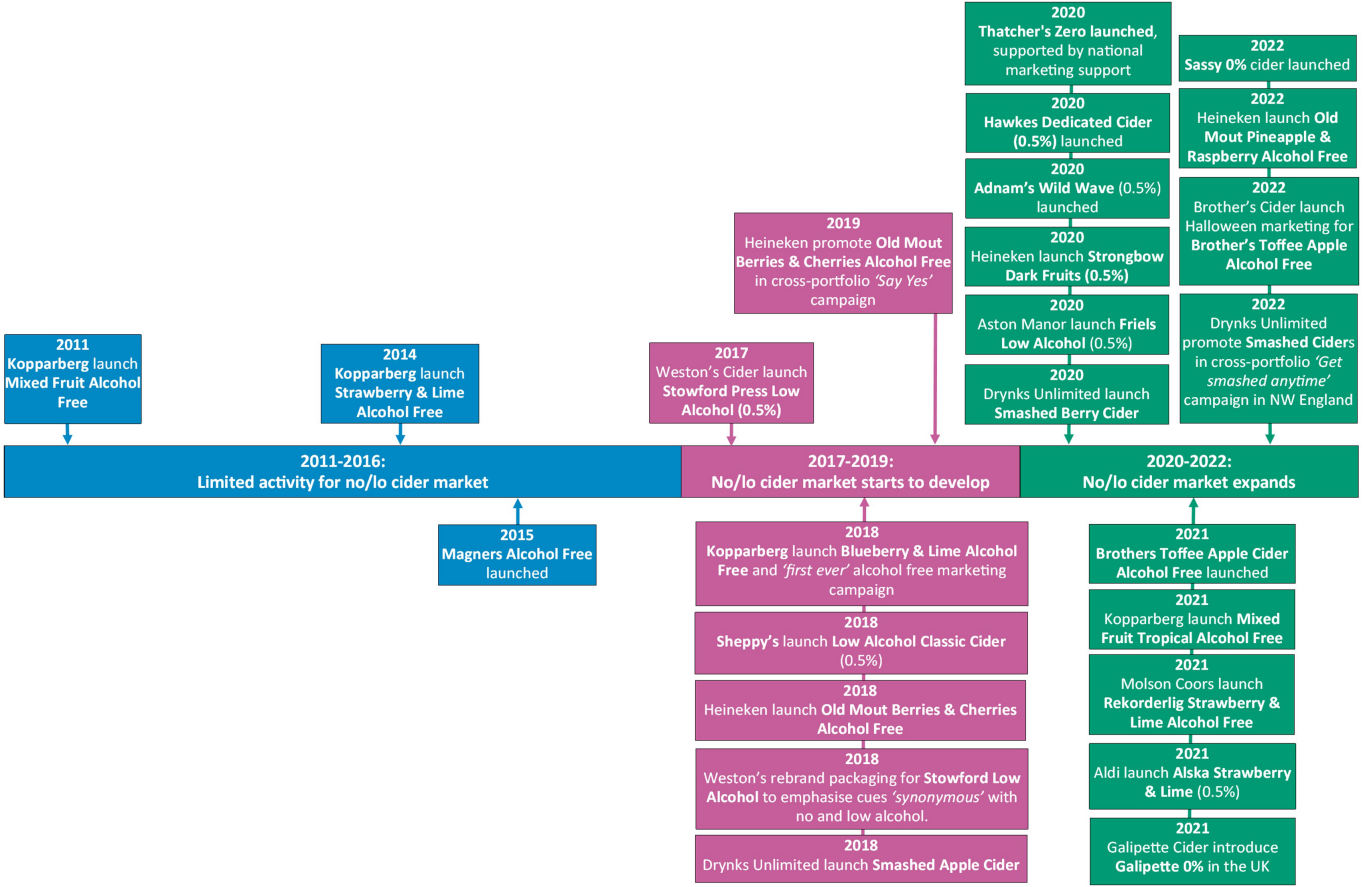
Timeline of key phases and example events in the development of the no/lo cider market between 2011 and 2022.

At time of writing, cider is the second largest sector in the no/lo market in GB, accounting for ~11% of sales value in 2023 [3]. Figure 3 illustrates the key phases and example events observed in its development. There was limited activity in phase one (2011–2016), mostly driven by flavour developments from Kopparberg [77, 78], who had already launched an alcohol‐free cider in 2010 [78]. There was some focus on lower‐ABV ciders, such as the launch of two 2.8% ABV ciders from Bulmers [79], albeit interest in this lower‐alcohol sector did not appear to match the size and scope observed for beers around the same time.

In phase two (2017–2019), the no/lo cider market began to develop, including launches from mainstream brands. For instance, Weston's Cider launched Stowford Press Low Alcohol (0.5%) in 2017 [80] and redesigned the packaging in 2018 to emphasise cues considered ‘*synonymous*’ with the no/lo market [81]. Also in 2018, Heineken launched Old Mout Alcohol Free Berries and Cherries (0%) [82] and Sheppy's launched Low Alcohol Classic Cider (0.5%) [83], while Kopparberg launched another alcohol‐free variant, Blueberry and Lime [84], and reportedly launched their ‘*first ever*’ alcohol‐free marketing campaign [85].

In the final phase (2020–2022), the cider market continued to expand, including further launches and marketing from established alcohol producers such as Thatcher's [86], Aston Manor [87] and Brother's [88]. This phase also appeared to be characterised by continued interest in fruit‐flavoured alcohol‐free ciders, mirroring a trend popular in the standard strength cider market. For example, in 2021 alone, Brother's Cider launched an alcohol‐free version of Toffee Apple cider [88], Kopparberg launched an alcohol‐free version of Mixed Fruit Tropical cider [89] and Molson Coors launched a Strawberry and Lime variant of Rekorderlig [88].

### 3.4 Wine

**FIGURE 4:**
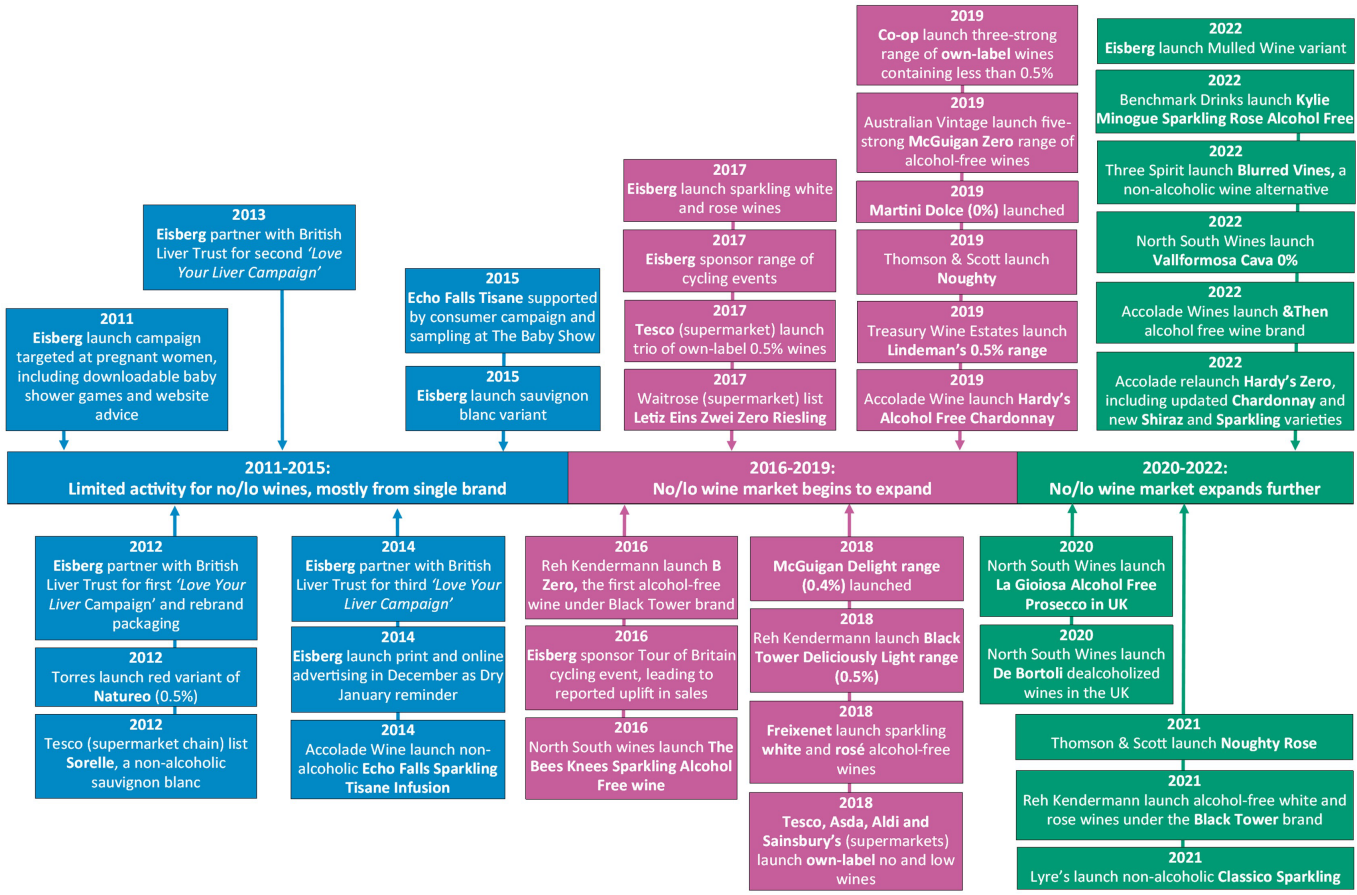
Timeline of key phases and example events reported in the development of the no/lo wine market between 2011 and 2022.

At time of writing, wine represents the third largest category in the no/lo market in GB, accounting for ~10% of sales value in 2023. [3]. Figure 4 illustrates the key phases and example events observed in its development.

During phase one (2011–2015), mainstream producers mostly focused on launching and marketing ‘lower‐strength’ offerings, typically around 5.5% ABV. This mirrors similar interest in lower‐ABV products in the beer category during this period, and likely also relates to pledges made in the PHRD [18, 19, 20] and the perceived ‘*favourable*’ tax breaks around this strength threshold [90, 91]. Activity for lower‐ABV wines involved many mainstream producers, including Accolade Wines [91], Australian Vintage [92], Brand Phoenix [93], E&J Gallo [94], Percy Fox (then‐subsidiary of Diageo) [95] and Reh Kendermann [96]. There was still some activity for no/lo wines, albeit mostly for the Eisberg brand. This included a marketing campaign aimed at pregnant women in 2011 [97], rebranded packaging in 2012 [98], an advertising campaign in December 2014 as a Dry January reminder [99], and a sauvignon blanc variant launched in 2015 [100]. Eisberg also partnered with the charity British Liver Trust for the ‘*Love Your Liver*’ campaign in January 2012, 2013 and 2014 [101, 102, 103].

The no/lo wine market began to emerge more in phase two (2016–2019), including launches from mainstream wine producers. For example, Reh Kendermann launched B Zero in 2016 [104], reportedly the first alcohol‐free wine under the mainstream Black Tower brand, and then extended their range to include Black Tower Deliciously Light (0.5% ABV) in 2018 [105]. Moreover, Australian Vintage launched the McGuigan Delight range (0.4% ABV) in 2018 [106] and launched five variants under the McGuigan Zero brand in 2019 [107]. Eisberg also used marketing to consolidate their position. For example, the brand's sponsorship of the Tour of Britain cycling event in 2016 reportedly contributed to a ‘*40% increase in sales year on year*’ [108] and stimulated further sponsorship of cycling races across 2017 [108].

The final phase (2020–2022) was characterised by consolidation and expansion among wine producers already active in the market, as well as some new entrants. Accolade Wine provide a key example of consolidation. They relaunched Hardy's Zero in 2022, including an updated and rebranded version of their Chardonnay product (originally launched in 2019 [109]) and new Shiraz and Sparkling varieties [110], and launched a new brand of alcohol‐free wines called & Then [111]. Accolade Wines highlighted that these new products were driven by ‘*revolutionary*’ [111] and ‘*cutting edge*’ [110] advancements in dealcoholisation technology, which they said now enabled them to remove the alcohol in a ‘*gentler*' manner, thus retaining ‘*more wine aromas and flavours without needing extra sugar*’ [112]. Despite these recent advances, however, it is noted that the no/lo wine market still has a much smaller share of the no/lo drinks market than standard wine does of the overall alcoholic drinks market.

### 3.5 Spirits

**FIGURE 5:**
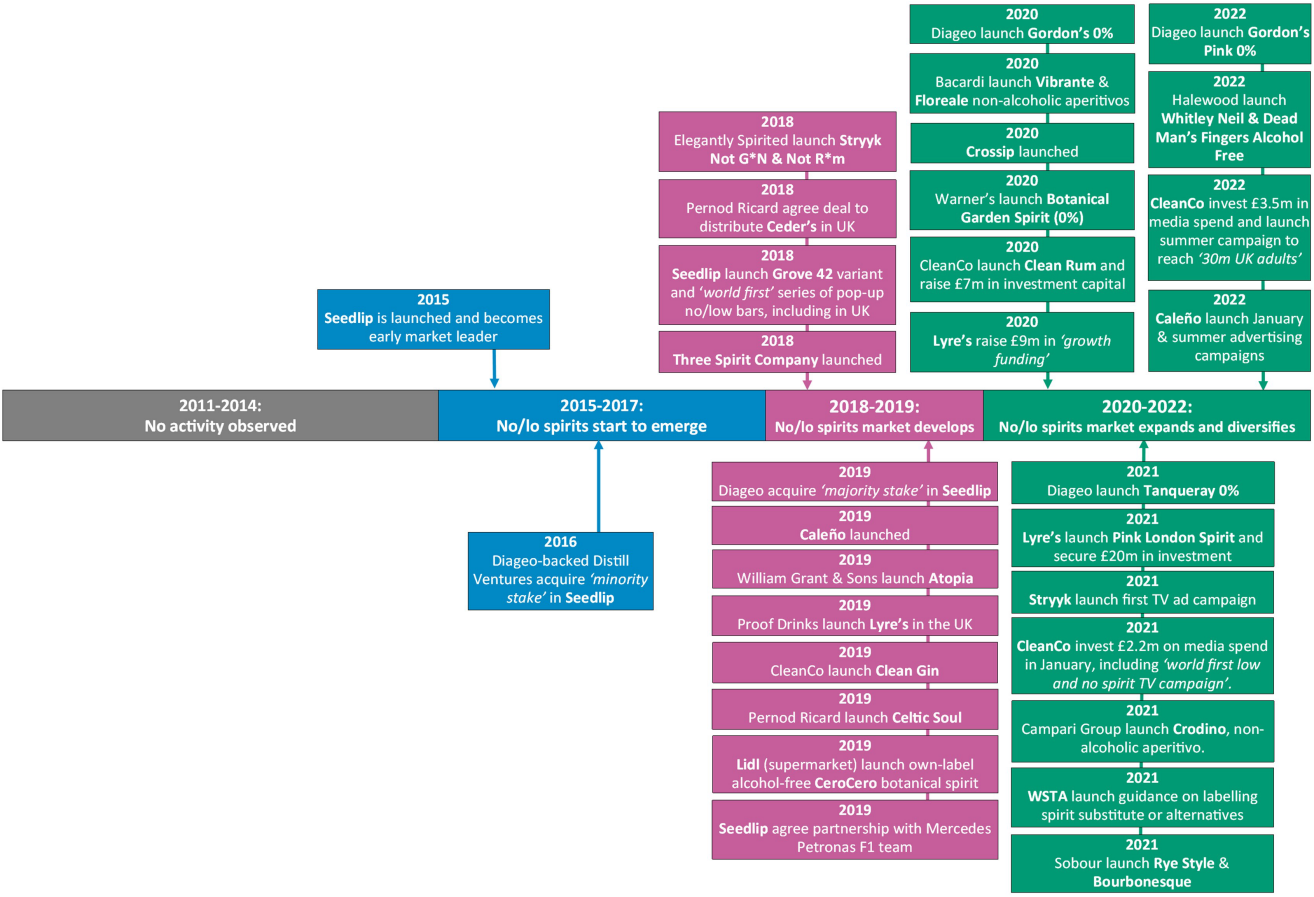
Timeline of key phases and example events reported in the development of the no/lo spirits market between 2011 and 2022.

The no/lo spirits market has emerged slower and later than other categories but still accounts for ~8% of sales value in 2023, a market share which is broadly comparable to cider and wine [3]. Figure 5 illustrates the key phases and example events observed in its development. In phase one (2015–2017), the only notable event was the 2015 launch of Seedlip [113], a distilled non‐alcoholic spirit drink with herb flavours. This brand became an early category leader and attracted investment from Diageo‐backed Distill Ventures, who acquired a minority stake in 2016 [114] and a majority stake in 2019 [115].

Phase two (2018–2019) was characterised by rapid expansion in the no/lo spirits market. Key launches included Ceder's and Celtic Soul from Pernod Ricard [16, 116], Stryyk Not G*n and Stryyk Not R*m from Elegantly Spirited [117], Caleño [118], Æcorn Aperitifs (a sister company to Seedlip) [119], Atopia from William Grant & Sons [16], Lyre's from Proof Drinks [16] and Clean Gin from The Clean Liquor Co (re‐named CleanCo in 2020 [120]). There was also evidence of Seedlip consolidating its market position, including through a reported ‘*world's first*’ series of pop‐up bars [121], launching new variants [122] and a partnership with the Mercedes AMG Petronas Motorsport team [16].

The final phase (2020–2022) was characterised by further market expansion and consolidation, including from mainstream producers. For example, Diageo launched an alcohol‐free version of Gordon's Gin in 2020 [123], which reportedly generated ~£1 million sales in its first month [124], and consolidated their position through the launch of an alcohol‐free version of Tanqueray Gin in 2021 [125] and a pink variant of Gordon's alcohol‐free in 2022 [126]. Similarly, in 2020, CleanCo consolidated and expanded their position by launching Clean Rum and raising £7 million of investment funding [120]. CleanCo also reportedly invested £2.2 million on media spend in January 2021, including what they claimed was ‘*the world's first low & no spirit TV campaign*’ [127], and followed this up with £3.5 million in media spend in 2022, including a summer marketing campaign reportedly reaching ‘*30 million UK adults*’ [128].

### 3.6 Ready‐to‐drink

**FIGURE 6:**
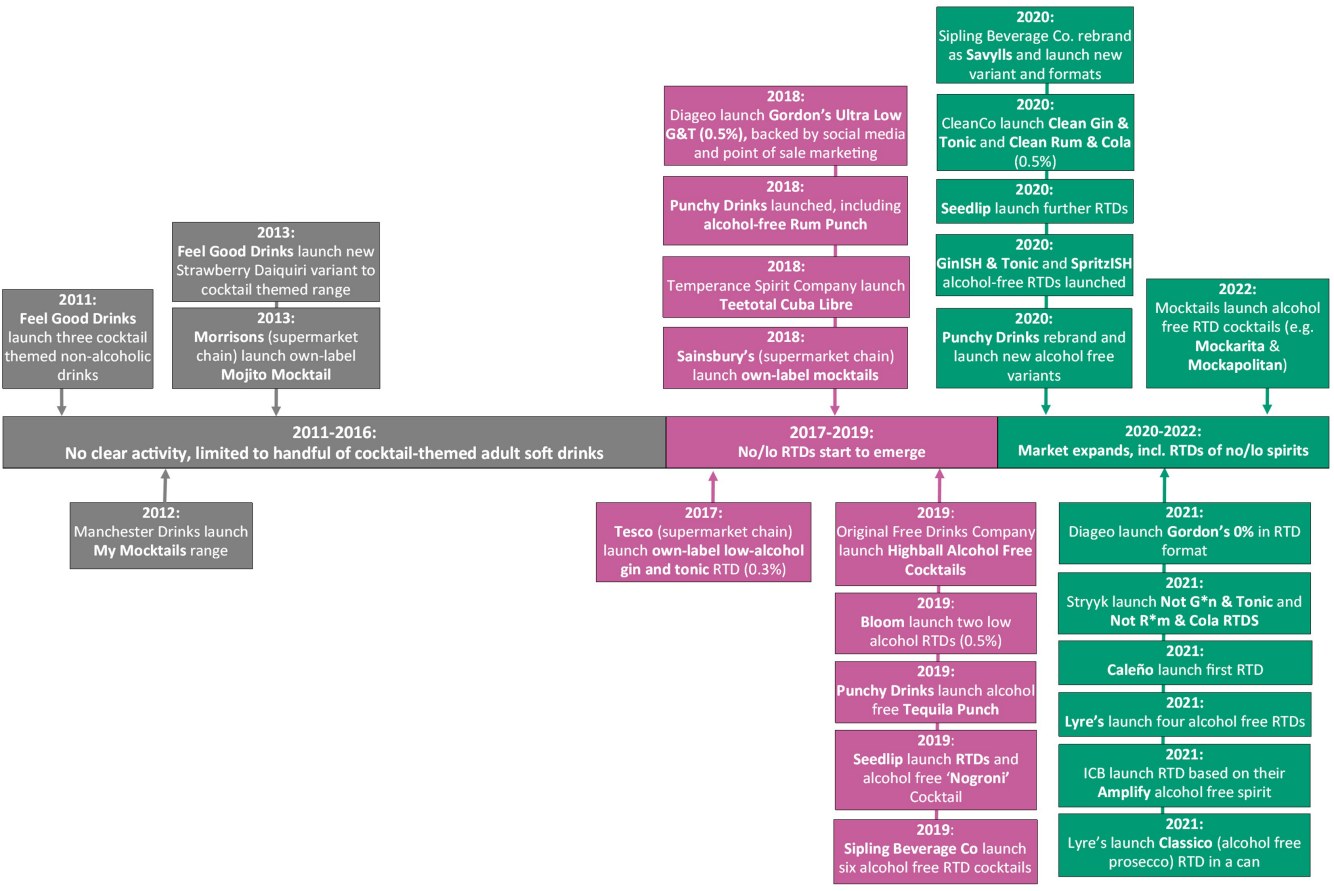
Timeline of key phases and example events reported in the development of the no/lo ready‐to‐drink (RTD) market between 2011 and 2022.

Figure 6 illustrates the key phases and example events observed in the development of the no/lo ready‐to‐drink market which, at the time of writing, accounts for only ~1% of no/lo sales value in GB [3]. There was no substantive activity in phase one (2011–2016), beyond some soft drink brands that sought to provide non‐alcoholic versions of popular cocktails [129, 130, 131]. However, there was evidence of proper market emergence in phase two (2017–2019), including Diageo's 2018 launch of Gordon's Ultra‐Low G&T (i.e., canned Gin and Tonic, 0.5% ABV), which was backed by social media and point‐of‐sale marketing [132], and the emergence of several independent companies specialising in no/lo ready‐to‐drink products, such as Punchy Drinks [16], Highball Cocktails from the Original Free Drinks Company [133] and Sipling Beverage Co (rebranded Savylls in 2020) [134]. The final phase (2020–2022) saw further market expansion, particularly product launches from brands that were already operating in the no/lo spirits market, such as CleanCo [120], Stryyk [135], Caleño [125] and Lyre's [125].

### 3.7 Adult soft drinks

Although adult soft drinks were not a primary focus of this study, they were often described as being closely related to, if not part of, the no/lo market. Table 2 provides examples of product launches which demonstrate this across the timeline. This activity included both larger producers of soft drinks and independent companies. For example, global soft drinks manufacturer Britvic created WiseHead Productions in 2016, an *‘incubator company*’ focused on ‘*zero‐proof*’ alcohol alternatives [136], while the smaller UK‐based company Belvoir Farms launched multiple fruit‐based products with alcohol connotations (e.g., Chardonnay Without the Hangover) [137, 138]. There was little evidence of alcohol companies moving into the soft drink market, except for BrewDog launching the sub‐brand POP Soda in 2022 [139].

**TABLE 2:** Example product launches in the adult soft drinks market that were presented as being related to, or interacting with, the alcohol‐free and low‐alcohol (no/lo) market.

Year	Launch and description
2011	SHS Drinks launch two limited‐edition products under the Shloer brand: Summer Fruit Punch, described as an *‘alcohol‐free Pimm's style’ drink'* ^a^ [146], and winter‐themed Berry Punch, marketed as *‘an alternative to alcohol’* [147]
2012	Halewood International launch Faith, a range of lightly carbonated soft drinks which were presented as *‘an alternative to alcohol’* and had variant names and product descriptions with wine connotations (e.g., sauvignon blanc, chardonnay, merlot) [148]
2013	SHS Drinks launch Shloer Celebration Pink Fizz and White Bubbly, which are described as ‘*premium non‐alcoholic fizz with a Champagne‐style cork and cage opening’* and delivering a *‘similar mouthfeel’* to Champagne [149]
2014	SHS Drinks launch Shloer Light, a lower‐calorie version of the grape‐based adult soft drink, in red grape and white grape varieties [150]
2015	Britvic launch J2O Spritz in three variants, supported by a £1.5 million marketing spend. Britvic said ‘*the gently sparkling drink…had a texture closer to sparkling wine or Champagne than a standard carbonated drink’* [151]
2016	Belvoir Fruit Farms launch their *‘without the hangover’* range of ‘*wine alternatives*’. This comprised three zero alcohol products which were based on popular wine types (e.g., Chardonnay Without the Hangover) [137]
2017	Soda Folk launch three craft soda flavours *‘inspired by booze’* (e.g., Juniper based on a gin & tonic) [152]
2018	Belvoir Fruit Farm launch a Botanical Juniper & Tonic, described as a *‘booze‐free G&T [gin & tonic] alternative’* [153]
2019	SHS launch Shloer Spritzed in three flavours, with the product reportedly *‘developed specifically as an alternative to the likes of gin & tonic’* [154]. The company also launch Shloer Pressed in three flavours, which was reportedly modelled on *‘the best‐performing fruit flavoured ciders’* [154]
2020	Belvoir Fruit Farms launch Non‐alcoholic Passionfruit Martini [155]
2021	Mighty Brew launch Organic Kombucha Elderflower Sec, described as an *‘alcohol‐free alternative to champagne and prosecco’* [156]
2022	BrewDog, primarily an alcohol producer, move into the soft drinks market with the POP Soda sub‐brand [139]

^a^ Pimm's is an English brand of gin‐based liqueur (25% abv).

It is also important to note that, during our observation period, the UK Government introduced a levy on soft drinks which contain added sugar. This policy, known as the Soft Drinks Industry Levy, was announced in March 2016 [140] and commenced in April 2018 [141]. The levy does not apply to alcoholic drinks (>1.2% ABV) and this exemption also extends to ‘*alcohol replacement drinks*’ which have been de‐alcoholised and are marketed and packaged like the drinks they intend to replace [142]. As such, this levy will plausibly have had a greater impact on the adult soft drinks market, where the methods of production do not involve dealcoholisation, versus the traditional no/lo categories described in earlier sections (e.g., no/lo beers, ciders, spirits or wines).

## 4 DISCUSSION

These narrative timelines provide key descriptive insight into the development of the no/lo market in GB to help inform and interpret research on no/lo drinks, such as analyses of consumer trends and market strategy. A key observation is that the no/lo market began to properly emerge and establish from 2015 onwards. While there was some activity prior to this, it was limited in scope and scale and largely did not involve established alcohol producers. From 2015 onwards, however, there was a shift in the frequency, intensity and volume of activity around no/lo drinks. This later period was characterised by intensive market entry, expansion, and consolidation among both independent producers of no/lo drinks and established producers of standard alcoholic drinks, the latter of whom focused on alcohol‐free products that shared branding with more mainstream products. Marketing campaigns also became larger, broader and more sustained in this period.

Importantly, our timelines suggest that the current no/lo drinks market emerged through a process of experimentation and exploration by producers and is likely still developing, with categories and individual companies moving at different speeds. In the latter years of the beer timeline, for example, there was evidence that some producers are still establishing their portfolios (e.g., AB InBev launching alcohol‐free versions of Stella Artois and Corona) while others are still optimising and strengthening their market position (e.g., Asahi UK replacing Peroni Libera with a core‐branded Peroni Nastro Azzurro 0% variant). Moreover, early activity in the spirits market was largely driven by companies who were not active in the standard‐strength market (e.g., Lyre's, Stryyk, CleanCo). It was only towards the end of the timeline that established spirits producers began to launch alcohol‐free alternatives of mainstream brands (e.g., Gordon's 0% and Tanqueray 0%), and these remain largely linked to gin, with comparatively less activity related to vodka, whiskey, or rum brands. It remains to be seen whether the number of spirit alternatives with standard alcohol branding continues to grow, as has been the norm for beers and cider. Finally, while activity for no/lo wines was observed throughout, reports towards the end of the timeline suggested that some producers have only recently acquired technology which enables them to make palatable no/lo wines in a cost‐effective manner, something considered a key barrier to market growth [143]. Collectively, this on‐going development suggests that we may not have yet reached a stable and mature no/lo market in GB that will be sustained in the longer term. From a public health perspective, this means that conclusions about the reach, size, and impact of the no/lo market, and the potential implications for consumption of standard‐strength alcohol or other public health outcomes, should be considered provisional and subject to longer‐term follow‐up once the market has matured.

Another observation is that the development of the no/lo drinks market has been predominately driven by supply‐side market forces, such as product launches, marketing campaigns, industry changes (e.g., acquisitions or investments) and retailer engagement (e.g., expanded ranges). There was also evidence that activity from third sector organisations is interacting with these market forces. For example, growing engagement in (temporary) abstinence during January has meant this month has become a focal period for new launches and marketing of no/lo drinks, while some brands were also named as partners of the official Dry January campaign [42]. Government activity was often only indirectly related to the no/lo drinks market, such as policy changes for standard‐strength alcoholic drinks and public awareness campaigns about drinking less alcohol. Even when the UK Government took direct actions towards no/lo drinks, this was limited to consulting on non‐mandatory guidance about descriptors [14, 15, 16] and a commitment to work with industry to grow the market [5], rather than any statutory policy interventions.

This review also highlights the importance of considering how the adjacent adult soft drinks market may interact with the no/lo market. Activity in this category ran concurrent to all stages of development of the no/lo market, and such products were often presented as competing for the same consumers and consumption occasions (see Table 2). Increased prominence of the no/lo drinks market may therefore have also increased the potential to promote certain soft drinks as part of that market, which may also lead to greater substitution between standard‐strength alcohol and soft drinks than was previously the case. Further research is needed into the increasing overlap between the alcohol, no/lo and soft drinks market, including at the corporate, marketing, policy and consumer level. For example, although outwith the observation period, Danish brewer Carlsberg has recently agreed to purchase Britvic, a leading soft drinks manufacturer and distributor in the UK [144].

To our knowledge, this is the first study to conduct an in‐depth assessment into development of the no/lo drinks market anywhere in the world. Key strengths include using industry‐focused data sources to generate novel insight into market development, observing trends over an extended period, analysing category‐specific trends, and considering trends in adjacent categories (e.g., lower alcohol and adult soft drinks). We also used an extensive range of sources to identify, triangulate and synthesise events, and manually reviewed all content to capture the diverse range of products, events and stakeholders involved in the market.

Nevertheless, there are limitations. We only sampled periodicals aimed at off‐trade retailers. This focus is likely to have captured most activity, as ~80% of no/lo sales volume in GB is through the off‐trade [3]. Nevertheless, future research should review publications aimed at the on‐trade (e.g., pubs/bars) to gain more detailed understanding about barriers and facilitators to development of no/lo in this market, such provision of no/lo drinks on draught and the use of no/lo spirits and adult soft drinks in made‐to‐order non‐alcoholic cocktails (‘mocktails’). Our analysis was also only limited to GB, and thus our observations about no/lo market development may not generalise to other jurisdictions. Similar research is needed to document trends in other countries with a growing no/lo drinks market, such as Australia [145], and to enable some degree of comparison about how the nature and timing of market development differs to GB.

We also only provide a descriptive and narrative summary of events. We do not critically appraise how the market and corporate political strategies of stakeholders have contributed to, emerged from, or adapted to market development. Specifically, while our analysis highlights the various products launched by companies, and highlights differing trends within and between categories, it does not examine strategic rationales for these actions (e.g., securing market share, responding to consumer trends) or efforts taken to shape the policy or market environment for no/lo drinks. Both aspects will be covered by separate parts of the overall project [13], drawing on both the data sources used here and other relevant sources (e.g., shareholder reports).

A final limitation is that our assessment about the significance of events was shaped by how they were framed in the data sources. Articles announcing product launches or marketing campaigns were understandably positive about anticipated appeal and reach. In some cases, articles provided quantifiable data to substantiate framing, such as the number of retailers listing the product or marketing expenditure and reach. In other cases, subsequent articles also provided detail which allowed us to assess whether the claims were realised. Frequently, however, articles did not contain such information, or events were not subject to follow‐up, and thus our assessment of importance is based on how they were described initially. Our analysis does, however, provide vital context to help inform the interpretation of both existing [2, 3, 4] and forthcoming studies [13] analysing how these events have impacted on sales, consumer use and preferences, and marketing activity.

In conclusion, this narrative review provides important descriptive insight into the emergence and development of the no/lo drinks market in GB between 2011 and 2022. The timelines highlight that the no/lo drinks market began to properly emerge and establish from 2015 onwards, with development largely driven by market factors such as product launches, marketing campaigns and retailer engagement. While initial development concentrated on beers, more recent developments in other categories (e.g., spirits and wines), coupled with continued consolidation and expansion in beers, suggest that the market may still develop further. As such, we recommend that any assessment of the public health impact of the no/lo drinks market should be considered provisional and subject to longer‐term follow‐up and validation once the market matures.

## AUTHOR CONTRIBUTIONS

Each author certifies that their contribution to this work meets the standards of the International Committee of Medical Journal Editors. The contribution of the individual authors is summarised as follows: *Conceptualisation*: JH, NF, NC. *Data curation*: NC, AM, KA, RH. *Formal analysis*: NC, AM. *Funding acquisition*: JH, NF. *Investigation*: NC, AM, KA, RH, NF, IK, JH. *Methodology*: NC, AM, KA, BH, IK. *Project administration*: NC. *Supervision*: JH, NF. *Visualisation*: NC. *Writing—original draft*: NC, AM. *Writing—review and editing*: NC, AM, KA, RH, NF, IK, JH.

## FUNDING INFORMATION

This research was funded by the National Institute for Health and Care Research (NIHR) Public Health Research Programme (NIHR135310). The views expressed are those of the author(s) and not necessarily those of the NIHR or the Department of Health and Social Care.

## CONFLICT OF INTEREST STATEMENT

NC was on the board of directors at Alcohol Focus Scotland between 2017 and 2022. All other authors have no conflicts of interest.

## Data Availability

Copies of the retail trade press magazines used in this review are available from their original publishers. The articles may also be available through online databases which capture these publcations. Copies of the market intelligence reports used in this review can be purchased directly from Mintel.
